# Benzylidene insertion reactions in organoplatinum chemistry: mechanism and selectivity

**DOI:** 10.1039/d6ra02340a

**Published:** 2026-04-21

**Authors:** Mohamed E. Moustafa, Paul D. Boyle, Richard J. Puddephatt

**Affiliations:** a Department of Chemistry, University of Western Ontario London N6A 5B7 Canada pudd@uwo.ca

## Abstract

The reaction of the cycloneophylplatinum complex [Pt(CH_2_CMe_2_C_6_H_4_)(NN)], 1, NN = 4,4′-di-*t*-butyl-2,2′-bipyridine (bubipy) or 2, NN = 3,4,7,8-tetramethyl-1,10-phenanthroline (phen*), with PhCHBr_2_ gave the corresponding complex [PtBr_2_(CH_2_CMe_2_C_6_H_4_CHPh)(NN)], 3 or 4, which are formed by formal oxidative insertion of benzylidene, PhCH, into the arylplatinum bond of 1 or 2 respectively. By monitoring the reactions by ^1^H NMR spectroscopy, intermediates were detected and shown to be products of oxidative addition [PtBr(CHBrPh)(CH_2_CMe_2_C_6_H_4_CHPh)(NN)], 5 and 6, along with a side product [PtBr_2_(CH_2_CMe_2_C_6_H_4_)(NN)], formed by formal bromine addition to 1 or 2. Oxidative addition also occurs with complex [PtMe_2_(bubipy)] to give a single isomer of [PtBrMe_2_(CHBrPh)(bubipy)]. The formation of 3 and 4 gives the first examples of selective benzylidene insertion reactions into aryl–platinum bonds, and mechanistic studies show that the reactions are retarded in the presence of LiBr. DFT calculations predict that the insertion reaction occurs synchronously or immediately following cleavage of the C–Br bond of intermediate 5 or 6, perhaps involving a shortlived benzylidene complex of platinum(iv). The potential for applications in catalysis of related reactions involving benzylidene insertion step is discussed.

## Abstract 2

The reaction of the cycloneophylplatinum complex [Pt(CH_2_CMe_2_C_6_H_4_)(NN)], 1, with PhCHBr_2_ gave the corresponding complex [PtBr_2_(CH_2_CMe_2_C_6_H_4_CHPh)(NN)].

## Graphical abstract

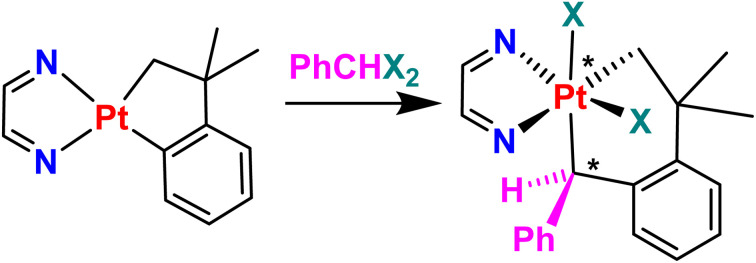

## 1 Introduction

Insertion reactions of alkenes or carbon monoxide into metal–alkyl or metal–aryl bonds leading to carbon–carbon bond formation are vital steps in many catalytic reactions, and have been studied for many years.^1^ More recently, insertion reactions of carbenes into metal–carbon bonds have attracted attention for their potential in catalysis, and diazoalkane or ylide reagents have often been studied as precursors to the carbene unit.^2–6^ Methylene dihalides have also been used as precursors for methylene insertion reactions.^2–9^ For example, oxidative addition of CH_2_X_2_ to complex 1 or 2 gave the corresponding organoplatinum(iv) complex *A*, followed by formal methylene insertion into the aryl–platinum bond to give *C*. A complication was that the free radical mechanism of oxidative addition led to the formation of some of the halogen adduct *B*.^9,10^

There are very few examples of insertion reactions of substituted carbenes into platinum–carbon bonds.^11–13^ A unique and elegant example in organoplatinum(iv) chemistry is shown in Scheme 2, in which the carbene *E* was obtained by protonation of a vinylplatinum precursor, *D*.^11^ The methylcarbene combined with a methylplatinum group to give the propene–platinum(ii) complex *F*, but it was not determined if the C–C bond forming step occurred by ethylidene insertion or in a reductive elimination reaction following proton transfer.^11^

There are no examples of benzylidene complexes of platinum though they are well established for many other transition metals,^14–19^ and a recent report suggests a benzylidene complex as a shortlived intermediate in cyclopropanation of alkenes.^20^ Complexes M=CHPh are involved in several important catalytic reactions, including alkene metathesis and polymerization.^14–20^ This article reports a clear example of effective benzylidene insertion into a Pt(IV)–C bond by using α,α-dibromotoluene, PhCHBr_2_, as reagent. A preliminary report of part of the work has been published, but this full report gives two further examples of reactions of organoplatinum(ii) complexes with benzylidene bromide and also reports experimental and theoretical studies of the reaction mechanisms involved.^21^

## 2 Results and discussion

The reaction of complex 1 with a 2-fold excess of benzylidene dibromide for five days led to precipitation of crystals of the product 3 (Scheme 3), which was isolated in 73% yield. The formation of complex 3 involves oxidation of the platinum(ii) center in 1 to platinum(iv) and both regioselective and stereoselective insertion of the benzylidene unit into the arylplatinum bond of the cycloneophyl group. Complex 3 contains two chiral centers (at PhHC* and Pt*), with descriptors *R*,*S* and *C*,*A* defining the chirality at carbon and platinum respectively,^22^Scheme 3, but only the *R-C*, *S-A* pair of enantiomers was formed. For example, in the ^1^H NMR spectrum of 3, only one singlet resonance was observed for the PtC*H*Ph proton at *δ* 5.71 (^2^*J*_PtH_ = 98 Hz) and one AB doublet was observed for the PtCH^A^H^B^ protons at *δ* 4.76 (^2^*J*_HH_ = 10 Hz and ^2^*J*_PtH_ = 102 Hz) and at *δ* 3.15 (^2^*J*_HH_ = 10 Hz and ^2^*J*_PtH_ = 88 Hz). None of the alternate *R-A*, *S-C* pair of enantiomers was detected, and no geometrical isomers of 3 or products of insertion into the Pt–CH_2_ bond of complex 1 were detected. The reaction of PhCHBr_2_ with complex 2 occurred in a similar way, again with very high selectivity to give complex 4 (Scheme 3).

**Fig. 1:**
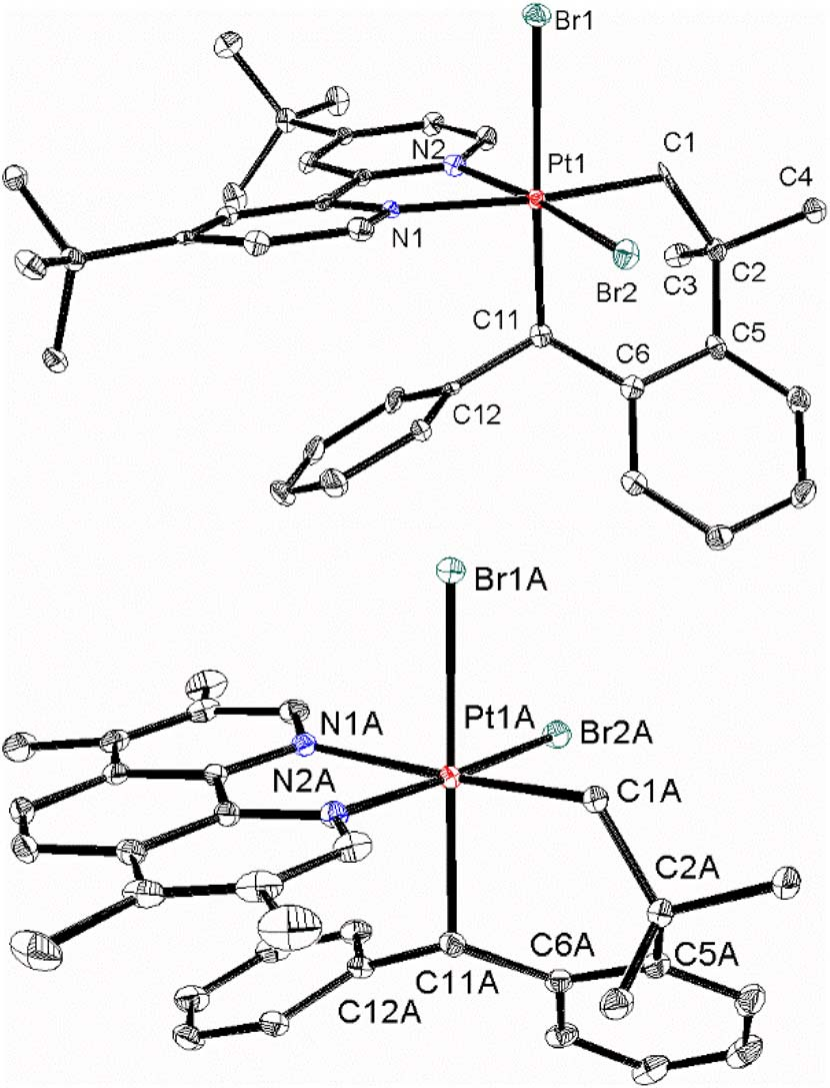
The structures of (above) complex 3, as the *R-A* enantiomer, and (below) one of the two independent molecules of complex 4, as the *S-C* enantiomer.

The NMR spectra show the broad structural features and high selectivity of the reactions but they do not define the precise structure of complex 3 or 4, so these were determined crystallographically (Fig. 1). Complex 3 crystallized in the chiral space group *P*2_1_2_1_2_1_ and the chosen crystal contained only the *R-A* enantiomer. In contrast, complex 4 crystallized in space group *P*1̄, with two independent molecules, and the crystal contained both symmetry equivalent *R-A* and *S-C* pairs of enantiomers of each. The structure determinations further define the geometric isomer as having a *cis*-PtBr_2_ unit and with the CH_2_ group *trans* to nitrogen and the CHPh group *trans* to bromide (Fig. 1).

**Fig. 2:**
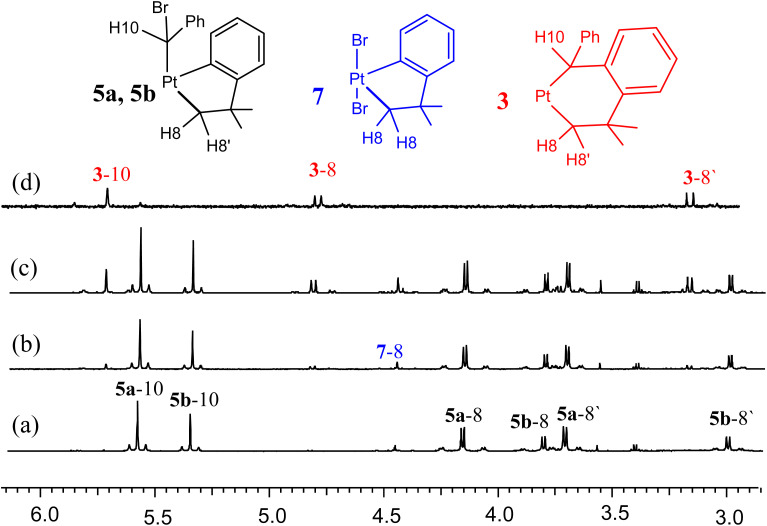
Monitoring of the reaction of PhCHBr_2_ with complex 1 by ^1^H NMR (acetone-d_6_, 600 MHz), showing the region of the PtC*H*Ph and PtC*H*_2_ protons only. The atom labels are defined in the partial structures above. Spectrum (a) after 2 h., mostly 5a and 5b with some 7; (b) after 4 h; (c) after 1 day; (d) spectrum of isolated product 3 (400 MHz).

In order to determine the reaction sequence leading to complex 3, the reaction of complex 1 with PhCHBr_2_ was monitored by ^1^H NMR spectroscopy. The reaction occurred according to Scheme 4 and selected spectra in the region of the PtC*H*Ph (H10) and PtC*H*_2_ (H8, H8′) resonances are shown in Fig. 2. The initial reaction occurred during an hour to give a mixture of two isomeric complexes [PtBr(CHBrPh)(CH_2_CMe_2_C_6_H_4_)(bubipy)], 5a and 5b, along with a lesser amount of *trans*-[PtBr_2_(CH_2_CMe_2_C_6_H_4_)(bubipy)], 7, which is an expected side product in a free radical oxidative addition mechanism. Complexes 5a and 5b are identified as two of the possible isomers of the C–Br oxidative addition of PhCHBr_2_. Over time, the resonances of 5a and 5b decayed while the resonances of the final product 3 grew (Fig. 2) until its concentration reached saturation and it began to precipitate from solution. The concentration of 7 remained roughly constant during the insertion reaction step represented in Scheme 4. Attempts to crystallize the intermediates 5a/5b were unsuccessful and the structures are not definitively defined. It is suggested that they are the most stable of the possible isomers of *trans* and *cis* oxidative addition. The ratio of 5a : 5b stayed roughly constant during the insertion reaction (Fig. 2), indicating either that they undergo insertion at about the same rate or, more probably, that they equilibrate with each other faster than undergoing insertion to form 3. In the ^1^H NMR spectra, each isomer of complex 5 gave a singlet resonance for the PtC*H*Ph proton [*δ* = 5.58, ^2^*J*_PtH_ = 44 Hz, for 5a; *δ* = 5.35, ^2^*J*_PtH_ = 42 Hz, for 5b] and two doublets for the PtC*H*_2_ protons [*δ* = 4.16, ^2^*J*_HH_ = 8 Hz, ^2^*J*_PtH_ = 100 Hz, and *δ* = 3.71, ^2^*J*_HH_ = 8 Hz, ^2^*J*_PtH_ = 70 Hz, for 5a; *δ* = 3.81, ^2^*J*_HH_ = 8 Hz, ^2^*J*_PtH_ = 98 Hz, and *δ* = 3.00, ^2^*J*_HH_ = 8 Hz, ^2^*J*_PtH_ = 73 Hz, for 5b] (Fig. 2). The presence of the aryl–platinum bond in 5a and 5b was shown by the observation of the coupling ^3^*J*_PtH_ for the *ortho* aryl proton [*δ* = 7.85, ^3^*J*_PtH_ = 21 Hz, in 5a; *δ* = 7.20, ^3^*J*_PtH_ = 28 Hz, in 5b], whereas the corresponding coupling ^4^*J*_PtH_ was too small to be resolved for the insertion product 3. A similar sequence of reactions was observed on reaction of complex 2 with PhCHBr_2_ to give intermediate isomers [PtBr(CHBrPh)(CH_2_CMe_2_C_6_H_4_)(phen*)], 6a and 6b, along with the known complex *trans*-[PtBr_2_(CH_2_CMe_2_C_6_H_4_)(phen*)], 8.^23^ However, the complexes were less soluble than those with the bubipy ligand, and only ^1^H NMR spectra of the intermediates could be obtained.

**Fig. 3:**
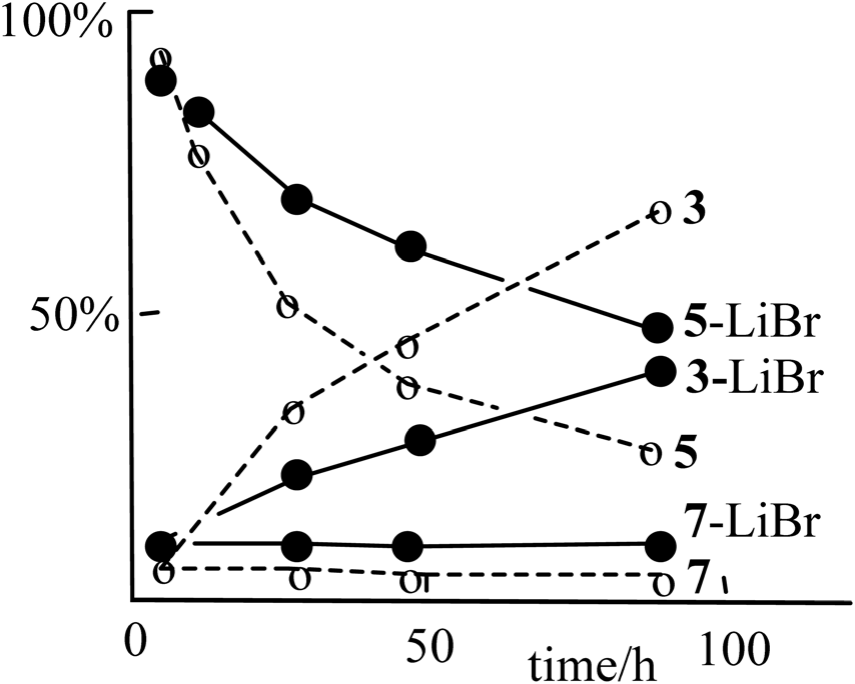
The effect of added lithium bromide on the rate of rearrangement of complex 5 (5a + 5b) to complex 3 in the presence (solid line) or absence (dashed line) of LiBr.

**Scheme 1:**
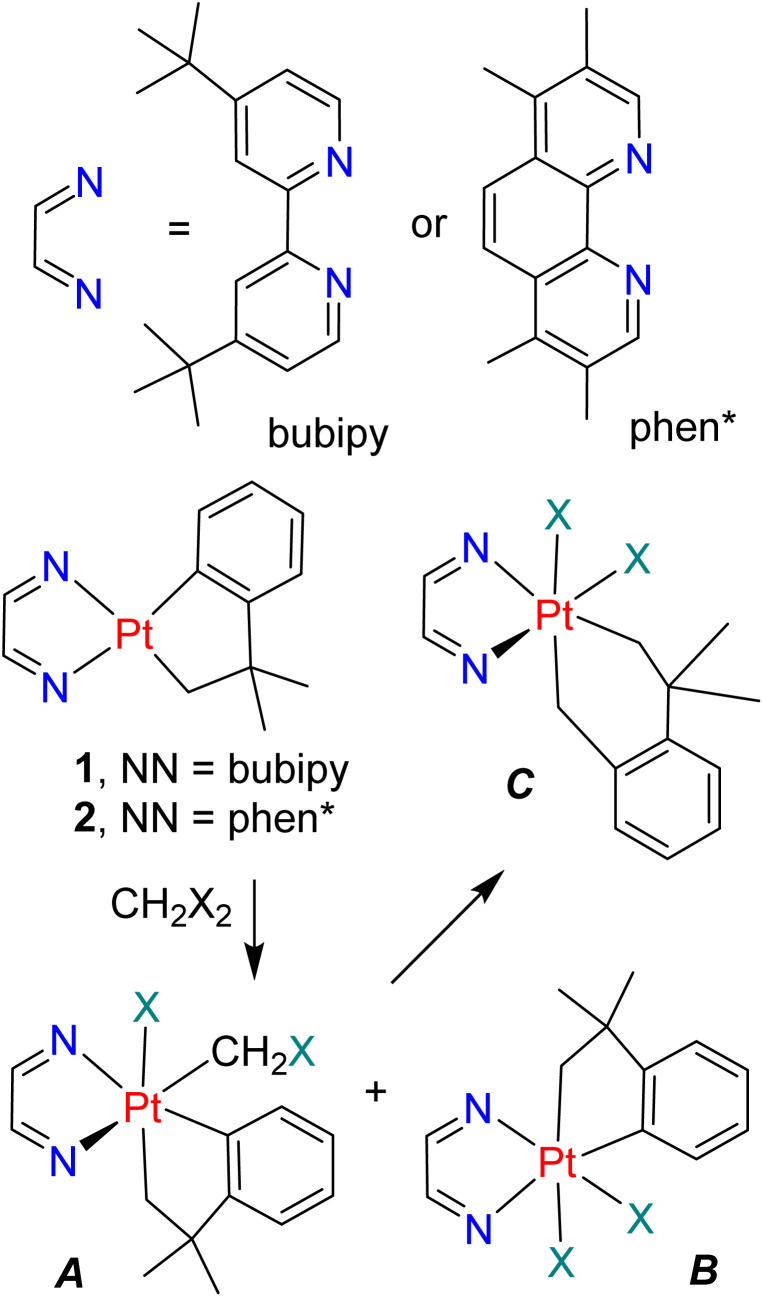
Carbene insertion following oxidative addition of CH_2_X_2_ (X = Cl, Br, I).

The effect of free bromide ion on the rate of the reaction was studied by carrying out parallel reactions of complex 1 with PhCHBr_2_ in acetone-*d*_6_ solution in the absence or presence of lithium bromide, with monitoring by ^1^H NMR spectroscopy (Fig. 3). There was no discernible effect on the oxidative addition step to form 5a and 5b, except for a slightly higher yield of the side product 7. However, the insertion step to form 3 was significantly retarded in the presence of LiBr. The effect was lower than in the related methylene insertion reaction of *A* to give *C* (Scheme 1, X = I), which was effectively prevented in the presence of lithium iodide.^9^

**Scheme 2:**
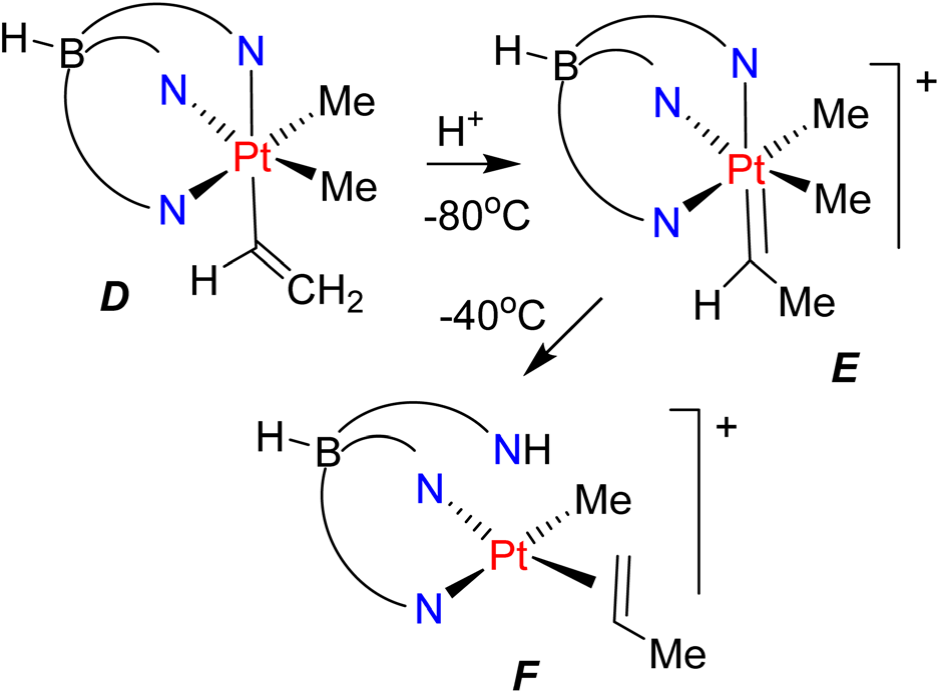
A possible example of ethylidene insertion in organoplatinum(iv) chemistry.

**Scheme 3:**
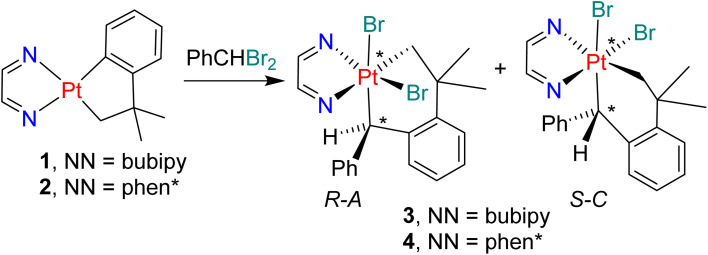
Benzylidene insertion from PhCHBr_2_. Chiral centers are marked *.

**Scheme 4:**
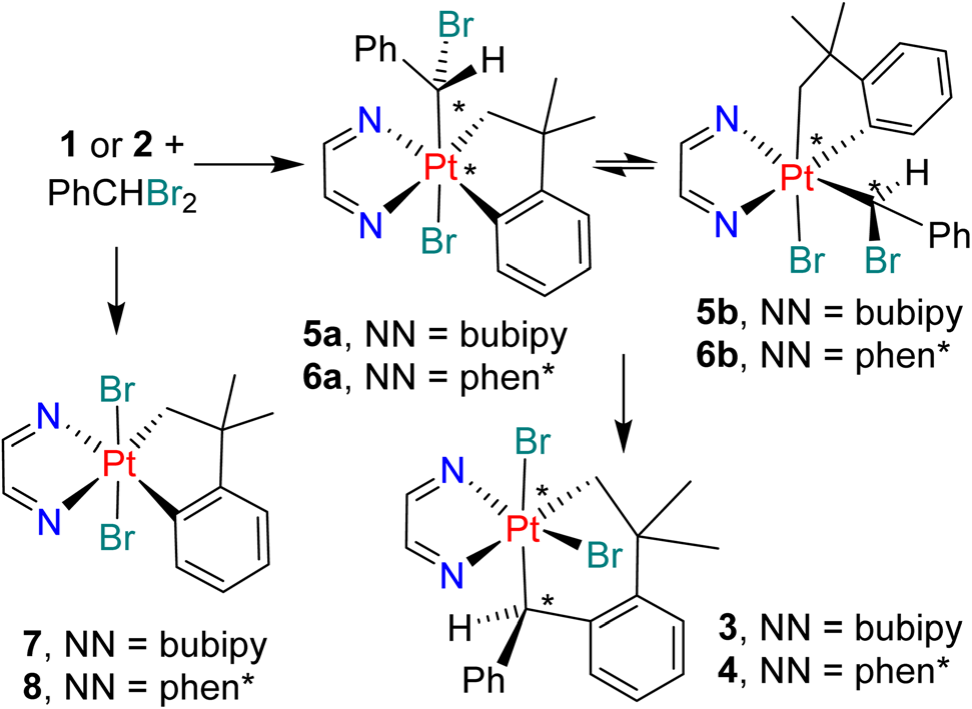
Intermediate and final products in the reaction of complex 1 with PhCHBr_2_ (NN = bubipy, chiral centers are indicated with * and only one enantiomer is shown in each case).

**Scheme 5:**
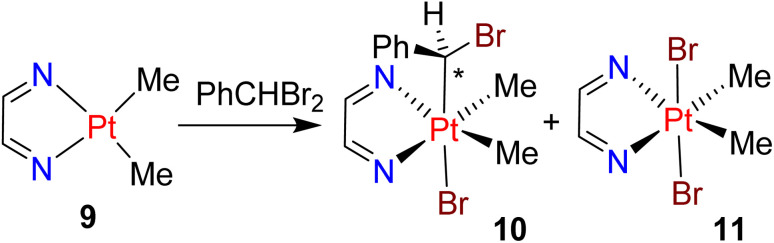
The reaction of [PtMe_2_(bubipy)], 9, with PhCHBr_2_.

The reaction of [PtMe_2_(bubipy)] with PhCHBr_2_ occurred rapidly and gave a mixture of the product of oxidative addition [PtBrMe_2_(CHBrPh)(bubipy)], 10, and the known bromine adduct *trans*,*cis*-[PtBr_2_Me_2_(bubipy)].^24,25^ Complex 10 was characterized in the ^1^H NMR spectrum by peaks for the PtC*H*BrPh proton at *δ* 5.23 with ^2^*J*_PtH_ = 48 Hz, and by two equal intensity methylplatinum resonances at *δ* 1.74 with ^2^*J*_PtH_ = 69 Hz, and at *δ* 1.46 with ^2^*J*_PtH_ = 69 Hz. Only one isomer 10 was observed in this case, and no subsequent insertion of benzylidene into a methylplatinum bond occurred after one day (Scheme 5).

**Scheme 6:**
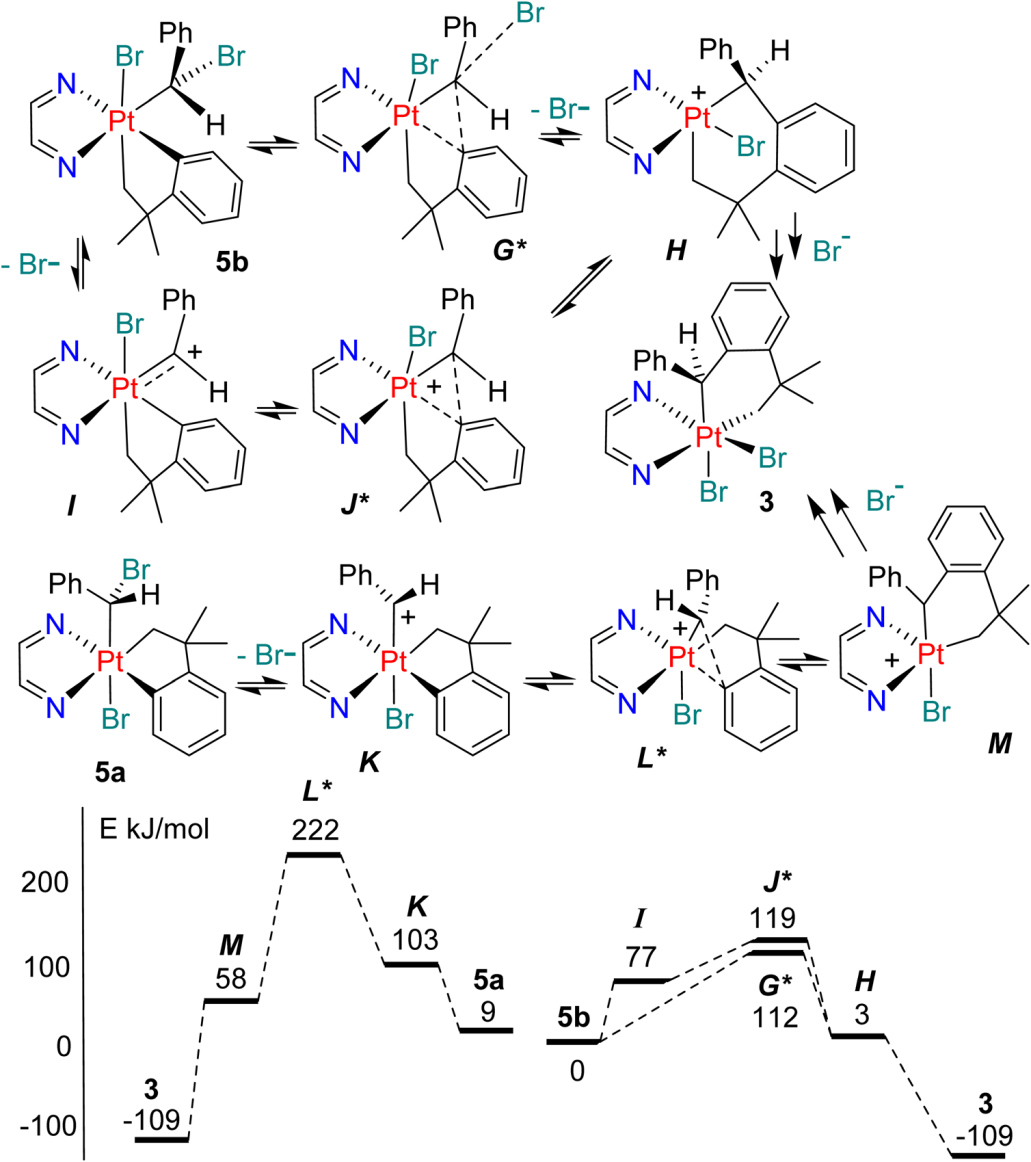
Some potential mechanisms of formation of the benzylidene insertion product 3 and the relative energies of intermediates and transition states (kJ mol^−1^) with respect to complex 5b.

**Fig. 4:**
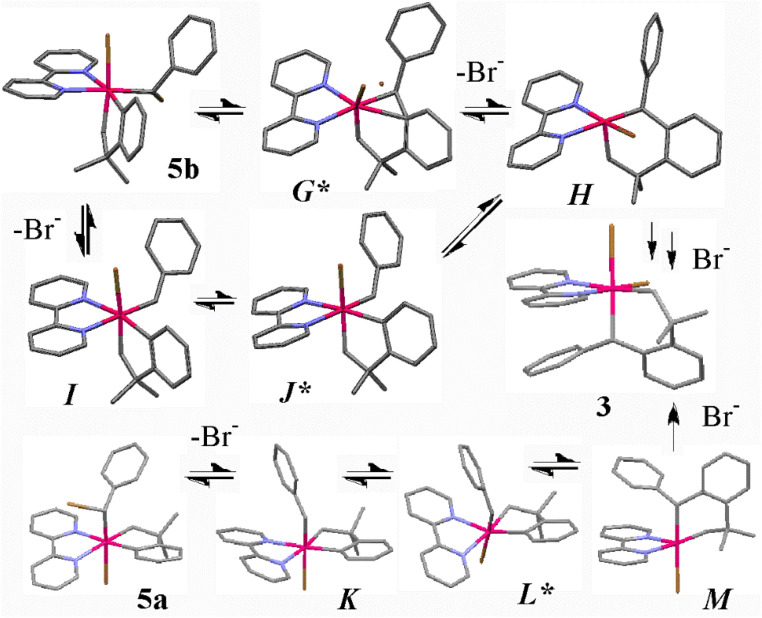
The calculated structures of the complexes of Scheme 6.

In order to gain insight into the mechanism of the benzylidene insertion reactions, some DFT calculations were carried out using the nudged elastic band (NEB) method to find transition states and COSMO to approximate solvation effects in dichloromethane solvent (using bipy as a substitute for the ligand bubipy or phen*, see experimental section for details).^26–31^ Previously, it was found that preliminary ionization from the simple halogenomethyl platinum complex [PtX(CH_2_X)(CH_2_CMe_2_C_6_H_4_)(bipy)] occurred most easily by dissociation of the Pt–X bond to give the transient cation [Pt(CH_2_X)(CH_2_CMe_2_C_6_H_4_)(bipy)]^+^ and that the insertion step and halide coordination followed sequentially to give [PtX_2_(CH_2_CMe_2_C_6_H_4_CH_2_)(bipy)].^9^ In contrast, the ionization energy from the complex [PtBr(CHPhBr)(CH_2_CMe_2_C_6_H_4_)(bipy)], 5a, is about equal for ionization of the Pt–Br or C–Br bond (111 or 103 kJ mol^−1^, respectively, with respect to 5b) and for the isomer 5b, the corresponding value for C–Br bond ionization of 77 kJ mol^−1^ is lowest to give the corresponding benzylidene complex [PtBr(=CHPh)(CH_2_CMe_2_C_6_H_4_)(bipy)]^+^, *I*, while the product of Pt–Br dissociation from 5b spontaneously rearranges to *I*. The differences from the simple bromomethylplatinum complexes probably arise from a combination of electronic stabilization of the cationic carbene *I* or *K* by the phenyl substituent aided by steric acceleration of bromide dissociation in 5b.^9^ The calculations predict that the key insertion step occurs during or after the bromide dissociation step from 5b (Scheme 6 and Fig. 4). Thus, the possible transition states G* and J* are both at considerably lower energy than the transition state L* arising from the *trans* isomer 5a (Scheme 6). Two mechanisms were considered, beginning with complex 5b. In the first, C–Br bond cleavage gives the cationic benzylidene complex, *I*, followed by insertion to give *H* by way of transition state J*. In the second, a more concerted process was considered in which the bromide dissociation and insertion steps were synchronous to give *H* by way of transition state G* (Scheme 6). In both cases, the 5-coordinate intermediate *H* must undergo pseudorotation, which is known to be facile,^32–35^ before bromide coordination occurs to give the observed product 3. The two insertion processes can be considered as intramolecular S_N_1 and S_N_2 mechanisms at the PtCHPhBr group of 5b, in which the leaving group is bromide and the nucleophile is the electron pair of the Pt–C_6_H_4_ bond.^36^ The more concerted reaction *via* transition state G* is slightly preferred, but the transition states G* and J* are similar in structure and energy and the Pt–Br bond is essentially fully dissociated in the transition state G* (calculated C–Br distance is 2.19 Å and 3.43 Å in 5b and G*, respectively).

## 3 Conclusions

The first examples of selective benzylidene insertion reactions into aryl–platinum bonds using the reagent PhCHBr_2_ with cycloneophylplatinum complexes [Pt(CH_2_CMe_2_C_6_H_4_)(NN)], with NN = bubipy or phen*, are established by structure determination of the products [PtBr_2_(CH_2_CMe_2_C_6_H_4_CHPh)(NN)]. Furthermore, the reactions are shown to occur by initial oxidative addition to give two isomers of intermediate complexes [PtBr(CHBrPh)(CH_2_CMe_2_C_6_H_4_CHPh)(NN)]. Oxidative addition also occurs with complex [PtMe_2_(bubipy)] to give a single isomer of [PtBrMe_2_(CHBrPh)(bubipy)], but no subsequent insertion occurred. The insertion step is subject to kinetic control and occurs selectively at the aryl Pt–C_6_H_4_ bond rather than the alkyl Pt–CH_2_ bond, but stereochemistry at platinum for both products of oxidative addition and insertion appears to be subject to thermodynamic control.

Monitoring of the benzylidene insertion reactions by ^1^H NMR spectroscopy in acetone-d_6_ solution shows that added LiBr does not greatly affect the rate of the oxidative addition step but inhibits the insertion step. DFT calculations predict that the insertion reaction occurs from the product of *cis* oxidative addition, and that it accompanies or follows cleavage of the C–Br bond of the initial product of oxidative addition. A shortlived benzylidene complex of platinum(iv) may be formed as a reaction intermediate. Known alkylidene complexes of platinum(iv) almost always have a heteroatom substituent, and these stable complexes are not known to undergo insertion reactions of the type observed in this work.^37–43^ Benzylidene complexes may be sufficiently stabilized by the phenyl substituent to be viable intermediates, while still being sufficiently reactive to give the key insertion step. The present work indicates that catalytic reactions involving benzylidene insertion steps, analogous to known reactions involving methylene insertion steps, have potential using PhCHBr_2_ as benzylidene source.^5^

## 4 Experimental

**Scheme 7:**
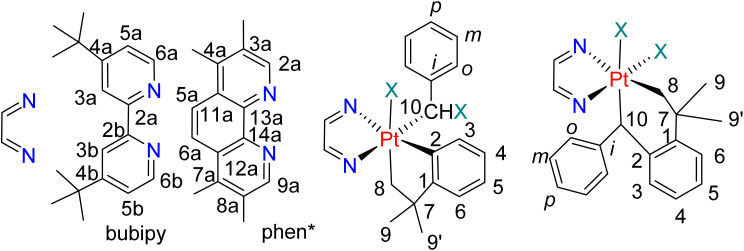
NMR labeling scheme.

NMR spectra were measured at 25 °C using Bruker Inova 400 and Inova 600 NMR spectrometers, with chemical shifts referenced to TMS. Peak assignments were confirmed with the aid of homo- and hetero-nuclear double resonance techniques (COSY, HSQC, HMBC), and the peak labeling system is given in Scheme 7.

Single-crystal X-ray diffraction measurements were made using a Bruker APEX-II CCD diffractometer with graphite-monochromated MoKα (*λ* = 0.71073 Å) radiation. Single crystals of the complexes were immersed in paraffin oil and mounted on MiteGen micromounts. The structures were solved using direct methods and refined by the full-matrix least-squares procedure of SHELXTL.^44–48^ Details are given in the cif files (CCDC 2132182–2132183). DFT calculations were carried out by using the Amsterdam Density Functional program based on the BP86 functional, with double-zeta basis set and first-order scalar relativistic corrections, as implemented in ADF 2019.^26–31^ Complexes 1 and 2 were prepared as described previously.^10^

### 4.1 [PtBr_2_(CH_2_CMe_2_C_6_H_4_CHPh)(bubipy)], 3

A solution of [Pt(CH_2_CMe_2_C_6_H_4_)(bubipy)], 1, was prepared *in situ* by stirring a solution of [Pt_2_(CH_2_CMe_2_C_6_H_4_)_2_(µ-SMe_2_)_2_] (0.050 g, 0.063 mmol) and bubipy (0.035 g, 0.130 mmol) in acetone (5 mL) for 30 min. To this solution was added α,α-dibromotoluene (43 µL, 0.26 mmol) and the mixture was stirred at room temperature for 5 days. The product, which precipitated from the solution, was collected by filtration, washed with pentane (2 × 5 mL), and dried under vacuum. Yield: 0.08 g, 73%. NMR in CD_2_Cl_2_: *δ*(^1^H) = 9.34 (d, 1H, ^3^*J*_HH_ = 6 Hz, ^3^*J*_PtH_ = 45 Hz, H6b), 9.32 (d, 1H, ^3^*J*_HH_ = 6 Hz, H6a), 7.94 (s, 1H, H3a), 7.79 (d, 1H, ^3^*J*_HH_ = 6 Hz, H5a), 7.58 (s, 1H, H3b), 7.46 (d, 1H, ^3^*J*_HH_ = 6 Hz, H5b), 7.41 (d, 1H, ^3^*J*_HH_ = 7 Hz, H3), 7.18 (t, 1H, ^3^*J*_HH_ = 7 Hz, H4), 7.10 (d, 1H, ^3^*J*_HH_ = 7 Hz, H6), 6.88 (t, 1H, ^3^*J*_HH_ = 7 Hz, H5), 6.82 (t, 1H, ^3^*J*_HH_ = 7 Hz, H*p*), 6.70 (m, 4H, H*o*, H*m*), 5.71 (s, 1H, ^2^*J*_PtH_ = 98 Hz, H10), 4.76 (d, 1H, ^2^*J*_HH_ = 10 Hz, ^2^*J*_PtH_ = 102 Hz, H8), 3.15 (d, 1H, ^2^*J*_HH_ = 10 Hz, ^2^*J*_PtH_ = 88 Hz, H8′), 1.76 (s, 3H, H9), 1.70 (s, 3H, H9′), 1.51 (s, 9H, *t*Bu),1.38 (s, 9H, *t*Bu). *δ*(^13^C) = 166.2 (C2a), 163.7 (C2b), 156.8 (C4a), 152.7 (C4b), 149.1 (6b), 148.3 (C6a), 147.9 (C2), 143.3 (C1), 142.1 (C*i*), 128.1 (C*o*,C*m*), 127.7 (C4), 126.3 (C3), 126.0 (C5,C6), 125.8 (C*p*), 125.1 (C5a,C5b), 121.8 (C3a), 119.5 (C3b), 39.4 (C7), 38.9 (C10), 36.3 (C-*t*Bu), 36.2 (C8), 35.9 (C-*t*Bu), 33.5 (C9), 31.9 (C9′), 30.6 (*t*Bu), 30.5 (*t*Bu).

### 4.2 [PtBr_2_(CH_2_CMe_2_C_6_H_4_CHPh)(phen*)], 4

To a solution of phen* (0.050 g, 0.21 mmol) in acetone (5 mL) was added [Pt_2_(CH_2_CMe_2_C_6_H_4_)_2_(µ-SMe_2_)_2_] (0.84 g, 0.11 mmol). The mixture was stirred for 1 h. At room temperature to give complex 2, then α,α-dibromotoluene (70 µL, 0.422 mmol) was added. The mixture was stirred for 2 days, then the yellow precipitate of the product was collected, washed with acetone (2 × 5 mL) and pentane (2 × 5 mL) and dried under vacuum. Yield: 0.136 g, 79%. NMR in CD_2_Cl_2_: *δ*(^1^H) = 9.49 (s, 1H, ^3^*J*_PtH_ = 41 Hz, H9a), 9.32 (s, 1H, ^3^*J*_PtH_ = 6 Hz, H2a), 8.09 (d, 1H, ^3^*J*_HH_ = 9 Hz, H5a), 8.02 (d, 1H, ^3^*J*_HH_ = 9 Hz, H6a), 7.58 (d, 1H, ^3^*J*_HH_ = 7 Hz, H3), 7.46 (d, 1H, ^3^*J*_HH_ = 7 Hz, H6), 7.39 (t, 1H, ^3^*J*_HH_ = 7 Hz, H4), 7.20 (t, 1H, ^3^*J*_HH_ = 7 Hz, H5), 7.15 (d, 2H, ^3^*J*_HH_ = 7 Hz, H*o*), 6.89 (t, 2H, ^3^*J*_HH_ = 7 Hz, H*m*), 6.53 (t, 1H, ^3^*J*_HH_ = 7 Hz, H*p*), 5.75 (s, 1H, ^3^*J*_PtH_ = 118 Hz, H10), 4.85 (d, 1H, ^3^*J*_HH_ = 11 Hz, ^3^*J*_PtH_ = 90 Hz, H8), 3.33 (d, 1H, ^3^*J*_HH_ = 11 Hz, ^3^*J*_PtH_ = 85 Hz, H8′), 2.86 (s, 3H, Me), 2.73 (s, 3H, Me), 2.63 (s, 3H, Me), 2.54 (s, 3H, Me), 1.82 (s, 3H, H9), 1.75 (s, 3H, H9′). *δ*(^13^C) = 150.8 (C2a), 149.3 (C9a), 148.9 (C7a), 148.1 (C1), 146.0 (C14a), 145.4 (C13a), 143.6 (C*i*), 143.5 (C4a), 142.8 (C8a), 142.1 (C2), 134.9 (C11a, C12a), 130.7 (C3a), 129.4 (C4), 128.1 (C*o*), 127.1 (C3), 126.3 (C5). 126.1 (C6), 125.8, (C*p*), 125.3 (C*m*), 125.2 (C5a), 123.4 (C6a), 39.8 (C7), 38.7 (^1^*J*_PtC_ = 500 Hz, C10), 36.1 (^1^*J*_PtC_ = 570 Hz, C8), 33.7 (C9), 32.0 (C9′), 19.0 (Me8a), 18.5 (Me3a), 16.0 (Me7a), 15.2 (Me4a).

### 4.3 [PtBr(CHBrPh)(CH_2_CMe_2_C_6_H_4_)(bubipy)], 5a and 5b, and *trans*-[PtBr_2_(CH_2_CMe_2_C_6_H_4_)(bubipy)], 6

The reaction of complex 1 with an equimolar amount of PhCHBr_2_ in acetone-d_6_ (1 mL), prepared as above, at room temperature was monitored by ^1^H NMR spectroscopy in the absence or presence of LiBr (1 equiv.). In the absence of LiBr, the reaction gave, after 4 h. Reaction time, complexes 5a, 5b, 6, and 3 in ratio 1 : 0.7 : 0.2 : 0.1. At this point, complete ^1^H and ^13^C NMR spectra were recorded. NMR in acetone-*d*_6_: 5a, *δ*(^1^H) = 8.86 (d, 1H, ^3^*J*_HH_ = 6 Hz, ^3^*J*_PtH_ = 12 Hz, H6a), 8.70 (s, 1H, 3a), 8.62 (s, 1H, 3b), 8.21 (d, 1H, ^3^*J*_HH_ = 6 Hz, ^3^*J*_PtH_ = 7 Hz, H6b), 7.91 (d, 1H, ^3^*J*_HH_ = 6 Hz, H5a), 7.85 (d, ^3^*J*_HH_ = 7 Hz, ^3^*J*_PtH_ = 21 Hz, H3), 7.38 (d, 1H, ^3^*J*_HH_ = 6 Hz, H5b), 6.99 (t, 1H, ^3^*J*_HH_ = 7 Hz, H4), 6.99 (t, 1H, ^3^*J*_HH_ = 7 Hz, H4), 6.92 (t, 1H, ^3^*J*_HH_ = 7 Hz, H5), 6.83 (m, 2H, H6,H*p*), 6.81 (t, 2H, ^3^*J*_HH_ = 7 Hz, H*m*), 6.33 (d, 2H, ^3^*J*_HH_ = 7 Hz, H*o*), 5.58 (s, 1H, ^2^*J*_PtH_ = 44 Hz, H10), 4.16 (d, 1H, ^2^*J*_HH_ = 8 Hz, ^2^*J*_PtH_ = 100 Hz, H8), 3.71 (d, 1H, ^2^*J*_HH_ = 8 Hz, ^2^*J*_PtH_ = 70 Hz, H8′), 1.59 (s, 3H, H9), 1.51 (s, 9H, *t*Bu), 1.46 (s, 3H, H9′), 1.44 (s, 9H, *t*Bu). *δ*(^13^C) = 165.8 (C1), 165.2 (C2a), 164.0 (C2b), 156.0 (C4a), 155.0 (C4b), 150.1 (C6a), 149.1 (C6b), 146.8, 134.2 (C*i*), 131.3 (C3), 130.5 (C5b), 127.8 (C*o*), 127.7 (C*m*, C*p*), 127.5 (C6), 125.7 (C5), 125.4 (C4), 124.6 (C5a), 121.7 (C3a), 121.5 (C3b), 45.2 (C8), 44.7 (C7), 40.3 (C10), 36.1(2 × C-*t*Bu), 34.9 (C9′), 34.7 (C9), 30.6 (2 × *t*Bu); 5b, *δ*(^1^H) = 9.61 (d, 1H, ^3^*J*_HH_ = 6 Hz, ^3^*J*_PtH_ = 7 Hz, H6a), 8.71 (s, 1H, 3a), 8.60 (s, 1H, 3b), 8.01 (d, 1H, ^3^*J*_HH_ = 6 Hz, H5a), 7.72 (d, 1H, ^3^*J*_HH_ = 6 Hz, ^3^*J*_PtH_ = 12 Hz, H6b), 7.28 (d, 1H, ^3^*J*_HH_ = 6 Hz, H5b), 7.20 (d, ^3^*J*_HH_ = 7 Hz, ^3^*J*_PtH_ = 28 Hz, H3), 6.98 (m, 3H, H4, H*o*), 6.91 (t, 1H, ^3^*J*_HH_ = 7 Hz, H5), 6.84 (d, 1H, ^3^*J*_HH_ = 7 Hz, H6), 6.83 (m, 1H, H*p*), 6.74 (t, 2H, ^3^*J*_HH_ = 7 Hz, H*m*), 5.35 (s, 1H, ^2^*J*_PtH_ = 42 Hz, H10), 3.81 (d, 1H, ^2^*J*_HH_ = 8 Hz, ^2^*J*_PtH_ = 98 Hz, H8), 3.00 (d, 1H, ^2^*J*_HH_ = 8 Hz, ^2^*J*_PtH_ = 73 Hz, H8′), 1.57 (s, 3H, H9), 1.52 (s, 9H, *t*Bu), 1.42 (s, 9H, *t*Bu), 1.38 (s, 3H, H9′). *δ*(^13^C) = 165.6 (C1), 164.9 (C2a), 164.4 (C2b), 155.9 (C4a), 155.3 (C4b), 150.9 (C6a), 146.8 (C6b), 134.7 (C*i*), 127.4 (C3), 127.3 (C*m*), 126.5 (C6), 125.3 (C*p*), 125.1 (C4), 125.0 (C5, C5a, C5b), 124.4 (C*o*), 121.8 (C3a), 121.7 (C3b), 43.9 (C8), 44.6 (C7), 37.2 (C10), 36.4 (C-*t*Bu), 36.3 (C-*t*Bu), 35.2 (C9), 34.4 (C9′), 30.7 (*t*Bu), 30.5 (*t*Bu). The spectra for 6 were identified by comparison to an authentic sample, prepared independently.

### 4.4 [PtBr(CHBrPh)(CH_2_CMe_2_C_6_H_4_)(phen*)], 6a and 6b

When the reaction of complex 2 with PhCHBr_2_ in acetone-*d*_6_ was monitored by ^1^H NMR spectroscopy, with COSY NMR to support assignments, the isomeric complexes [PtBr(CHBrPh)(CH_2_CMe_2_C_6_H_4_)(phen*)], 6a and 6b were detected at intermediate stages. NMR in CD_2_Cl_2_: 6a, *δ*(^1^H) = 5.69 (s, 1H, ^2^*J*_PtH_ = 42 Hz, H10), 4.33 (d, 1H, ^2^*J*_HH_ = 10 Hz, ^2^*J*_PtH_ = 110 Hz, H8), 3.86 (d, 1H, ^2^*J*_HH_ = 10 Hz, ^2^*J*_PtH_ = 66 Hz, H8′), 2.85 (s, 3H, Me), 2.79 (s, 6H, 2Me), 2.72 (s, 6H, 2Me), 1.68 (s, 3H, H9), 1.58 (s, 3H, H9′). 6b, *δ*(^1^H) = 5.41 (s, 1H, ^2^*J*_PtH_ = 40 Hz, H10b), 3.95 (d, 1H, ^2^*J*_HH_ = 10 Hz, ^2^*J*_PtH_ = 106 Hz, H8b), 3.09 (d, 1H, ^2^*J*_HH_ = 10 Hz, ^2^*J*_PtH_ = 66 Hz, H8b′), 2.86 (s, 3H, Me), 2.79 (s, 3H, Me), 2.71 (s, 3H, Me), 2.35 (s, 3H, Me), 1.67 (s, 3H, H9), 1.55 (s, 3H, H9′).

### 4.5 *Trans*-[PtBr_2_(CH_2_CMe_2_C_6_H_4_)(bubipy)], 7

A solution of [Pt(CH_2_CMe_2_C_6_H_4_)(bubipy)] was prepared *in situ* by the reaction of [Pt_2_(CH_2_CMe_2_C_6_H_4_)_2_(µ-SMe_2_)_2_] (0.050 g, 0.063 mmol) with bubipy (0.035 g, 0.130 mmol) in CH_2_Cl_2_ (5 mL). The reaction mixture was stirred briefly at room temperature then excess of Br_2_ (49 µL, 0.325 mmol) was added, the colour changed immediately from orange to yellow. The mixture was stirred for 1 h, then pentane (5 mL) was added to give the product as yellow solid, which was separated by filtration, washed with pentane (2 × 5 mL), and dried under vacuum. Yield: 0.079 g, 80%. NMR in CD_2_Cl_2_: *δ*(^1^H) = 9.38 (d, 1H, ^3^*J*_HH_ = 6 Hz, ^3^*J*_PtH_ = 11 Hz, H6a), 8.78 (d, 1H, ^3^*J*_HH_ = 6 Hz, ^3^*J*_PtH_ = 12 Hz, H6b), 8.28 (s, 2H, H3a,3b), 7.80 (d, 1H, ^3^*J*_HH_ = 6 Hz, H5a), 7.70 (d, 1H, ^3^*J*_HH_ = 6 Hz, H5b), 7.48 (d, 1H, ^3^*J*_HH_ = 8 Hz, ^3^*J*_PtH_ = 30 Hz H3), 7.02 (t, 1H, ^3^*J*_HH_ = 8 Hz, H4), 6.93 (t, 1H, ^3^*J*_HH_ = 8 Hz, H5), 6.84 (d, 1H, ^3^*J*_HH_ = 8 Hz, H6), 4.43 (s, 2H, ^2^*J*_PtH_ = 100 Hz, H8), 1.49 (s, 9H, *t*Bu), 1.48 (s, 9H, *t*Bu), 1.43 (s, 6H, H9).

### 4.6 [PtBrMe_2_(CHBrPh)(bubipy)], 10

To a solution of [PtMe_2_(bubipy)], 9, (0.05 g, 0.114 mmol) in acetone (5 mL) was added α,α-dibromotoluene (43 µL, 0.260 mmol). The mixture was stirred at room temperature for 2 h. and the yellow precipitate of the product was collected by filtration, washed with pentane (2 × 5 mL) and dried under vacuum. Yield: 0.064 g, 81%. NMR in dichloromethane-*d*_2_: *δ*(^1^H) = 8.93 (d, 1H, ^3^*J*_HH_ = 6 Hz, ^3^*J*_PtH_ = 13 Hz, H6a), 8.86 (d, 1H, ^3^*J*_HH_ = 6 Hz, ^3^*J*_PtH_ = 12 Hz, H6b), 8.18 (s, 1H, H3a), 8.13 (s, 1H, H3b), 7.66–7.73 (m, 2H, H5a, H5b), 6.78 (t, 1H, ^3^*J*_HH_ = 7 Hz, H*p*), 6.69–6.71 (m, 4H, H*o*, H*m*), 5.23 (s, 1H, ^2^*J*_PtH_ = 48 Hz, H10), 1.74 (s, 3H, ^2^*J*_PtH_ = 69 Hz, PtMe^a^), 1.51 (s, 9H, *t*Bu), 1.46 (s, 3H, ^2^*J*_PtH_ = 69 Hz, PtMe^b^), 1.40 (s, 9H, *t*Bu); *δ*(^13^C): 165.1 (C2b), 164.9 (C2a), 155.8 (C4a), 155.1 (C4b), 149.1 (C6a), 147.8 (C*i*), 147.7 (C6b), 128.0 (C*p*), 126.8 (C*m*), 125.6 (C*o*), 125.3 (C5a), 124.9 (C5b), 121.6 (C3a), 120.8 (C3b), 40.3 (*C*HBrPh), 36.6 (*C*Me_3_), 36.3 (*C*Me_3_), 31.3 (*t*Bu), 31.1(*t*Bu), 2.0 (Pt*Me*), 0.4 (Pt*Me*). When the reaction was monitored by ^1^H NMR spectroscopy, the products were identified as 10 and *trans*,*cis*-[PtBr_2_Me_2_(bubipy)], 11, identified by comparison with an authentic sample.^24,25^

## Conflicts of interest

There are no conflicts to declare.

## Supplementary Material

RA-016-D6RA02340A-s001

RA-016-D6RA02340A-s002

RA-016-D6RA02340A-s003

## Data Availability

CCDC 2132182 and 2132183 contain the supplementary crystallographic data for this paper.^49*a*,*b*^ Primary data for this paper are given in the supplementary information (SI) (NMR spectra of the compounds). Data from the DFT calculations are given in the file phchxyz.xyz. Crystallographic data have been deposited with the Cambridge Crystallographic data base. Supplementary information: NMR spectra of the complexes. The file phchxyz.xyz contains the calculated coordinates of ground and transition states of the complexes. See DOI: https://doi.org/10.1039/d6ra02340a.
