# Supplementary material for: Benzylidene insertion reactions in organoplatinum chemistry: mechanism and selectivity
Source: RSC Adv. 2026 Apr 21;16(23):20600–8. doi: 10.1039/d6ra02340a (PMC13098486; doi:10.1039/d6ra02340a)
Supplement: RA-016-D6RA02340A-s002 [file RA-016-D6RA02340A-s002.pdf]

Supplementary Information (SI) for RSC Advances.
This journal is © The Royal Society of Chemistry 2026

Benzylidene Insertion Reactions in Organoplatinum Chemistry: Mechanism and Selectivity

Mohamed E. Moustafa, Paul D. Boyle, Richard J. Puddephatt*

Department of Chemistry, University of Western Ontario, London, N6A 5B7, Canada

Supporting Information

Figures S1-S13: NMR spectra of the complexes

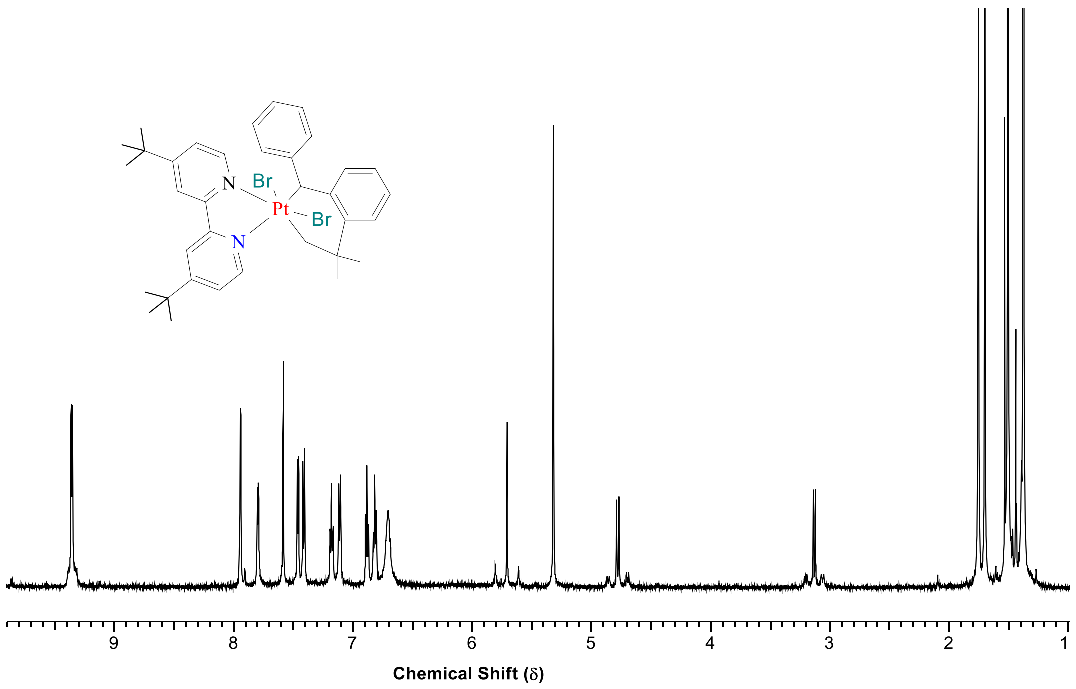

Figure S1: ^{1}H NMR spectrum of complex **3** in CD_2Cl_2.

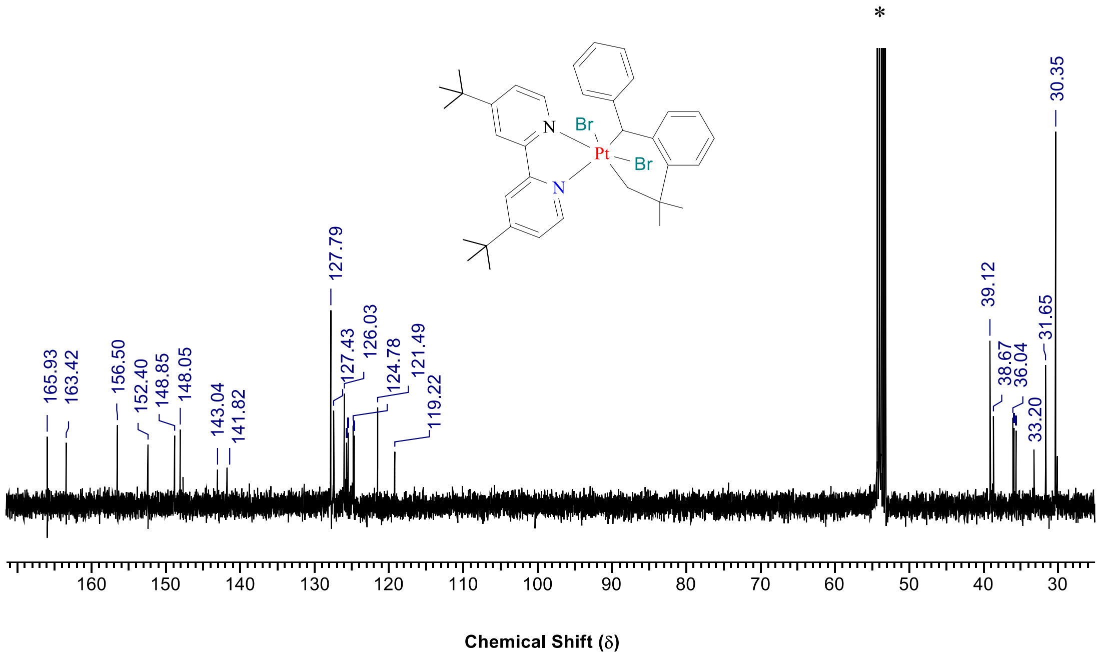

Figure S2: ^{13}C NMR spectrum of complex **3** in CD_2Cl_2.

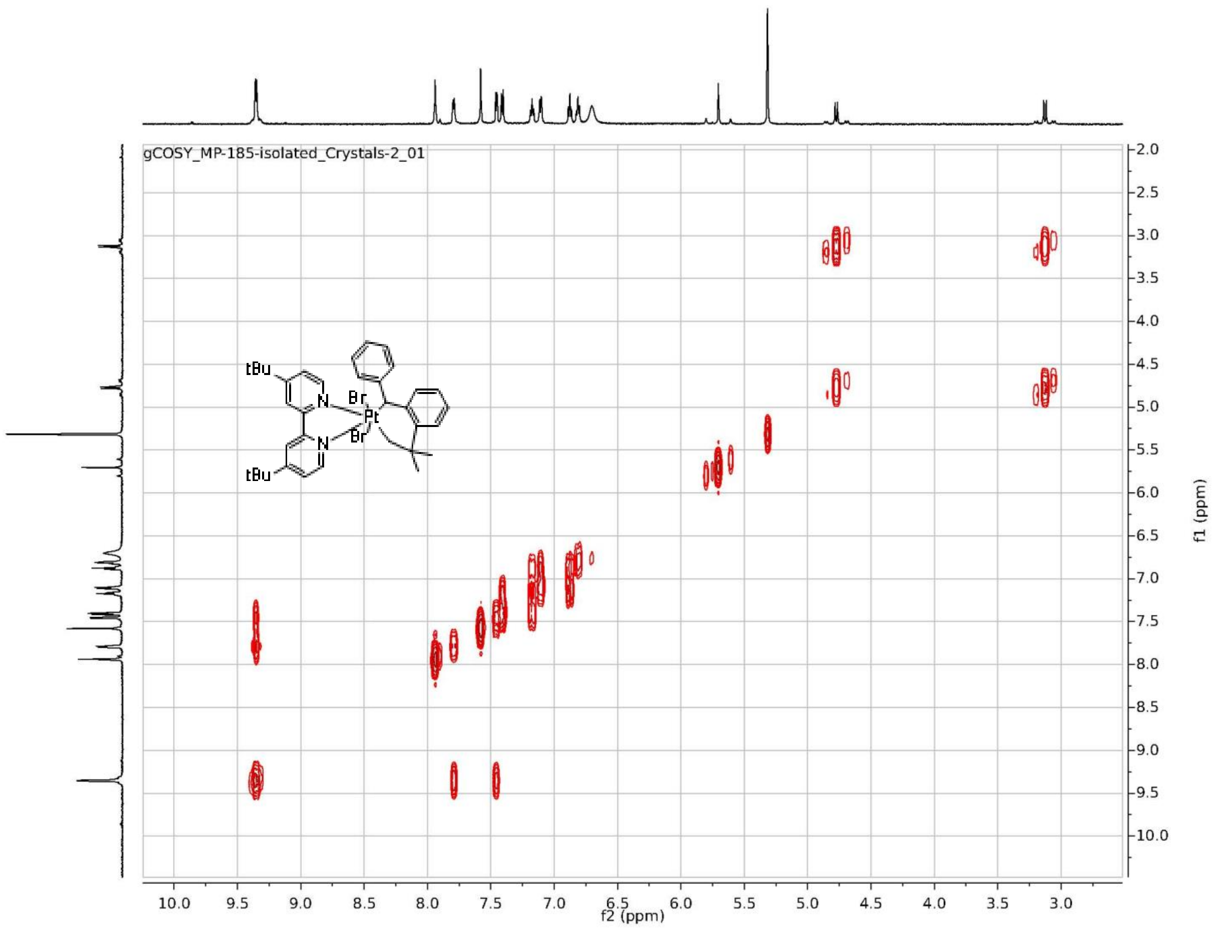

Figure S3: ^{1}H-^{1}H COSY spectrum of complex **3** in CD_2Cl_2.

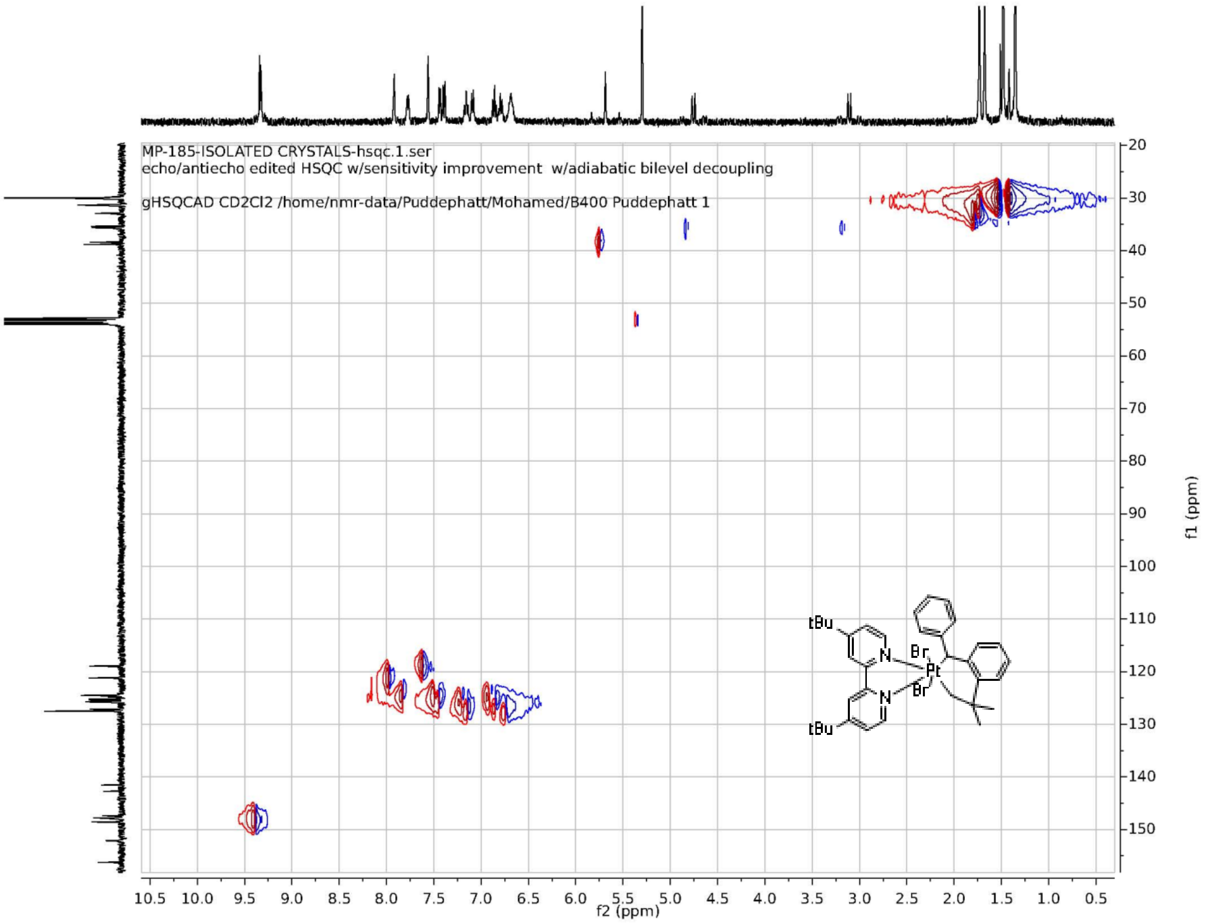

Figure S4: ^{1}H-^{13}C HSQC spectrum of complex **3** in CD_2Cl_2.

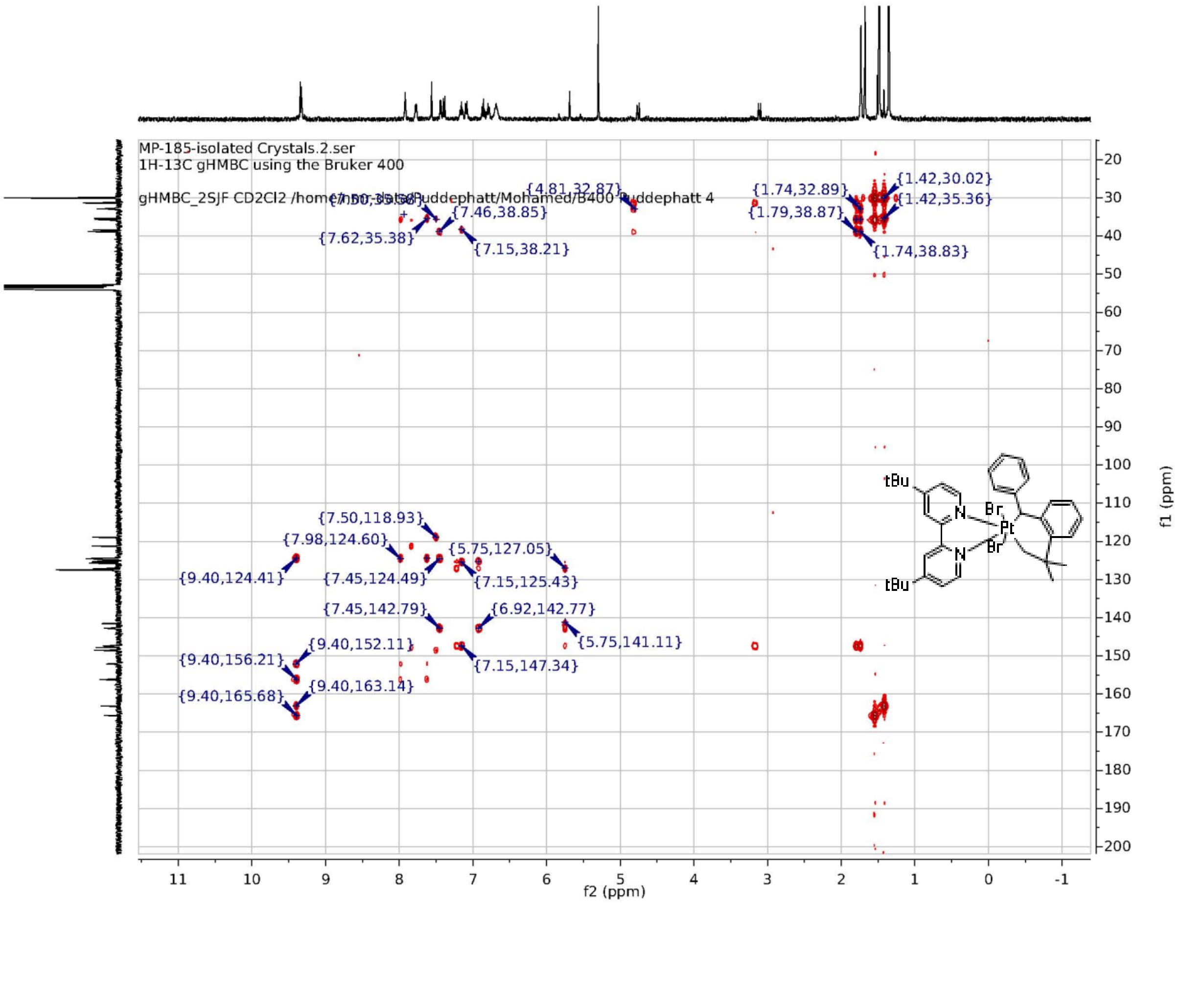

Figure S5: ^{1}H-^{13}C HMBC spectrum of complex **3** in CD_2Cl_2.

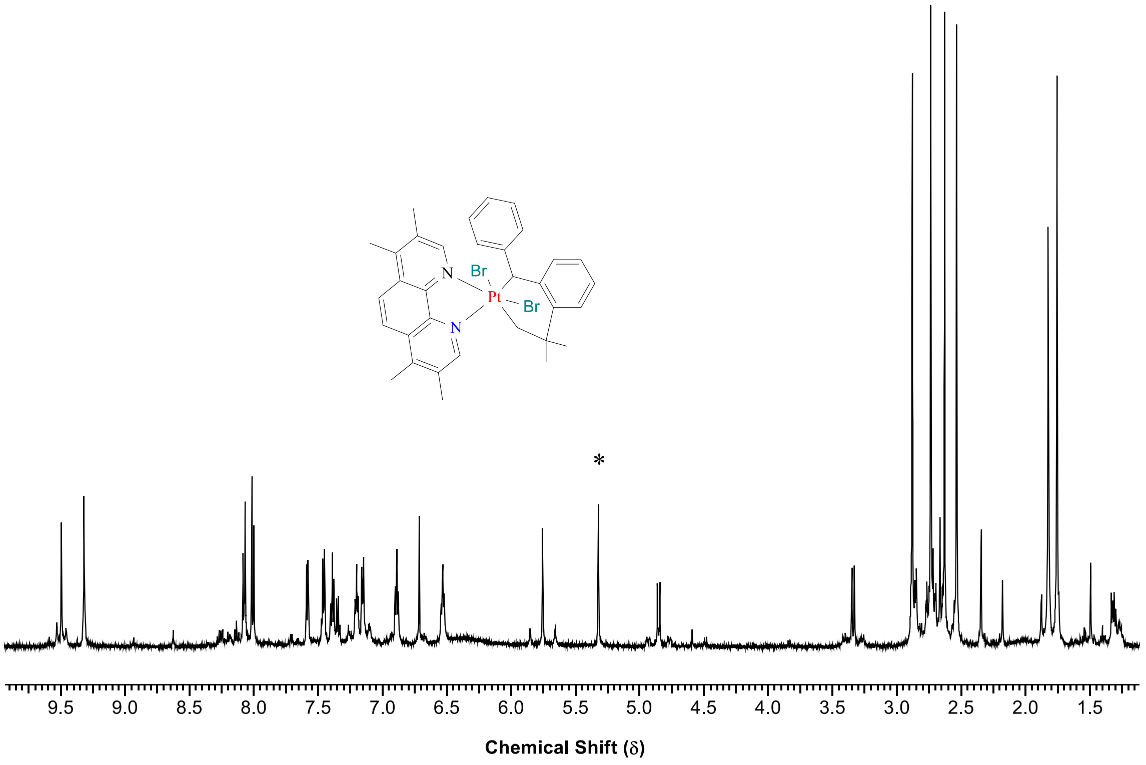

Figure S6: ^{1}H NMR spectrum of complex **4** in CD_2Cl_2.

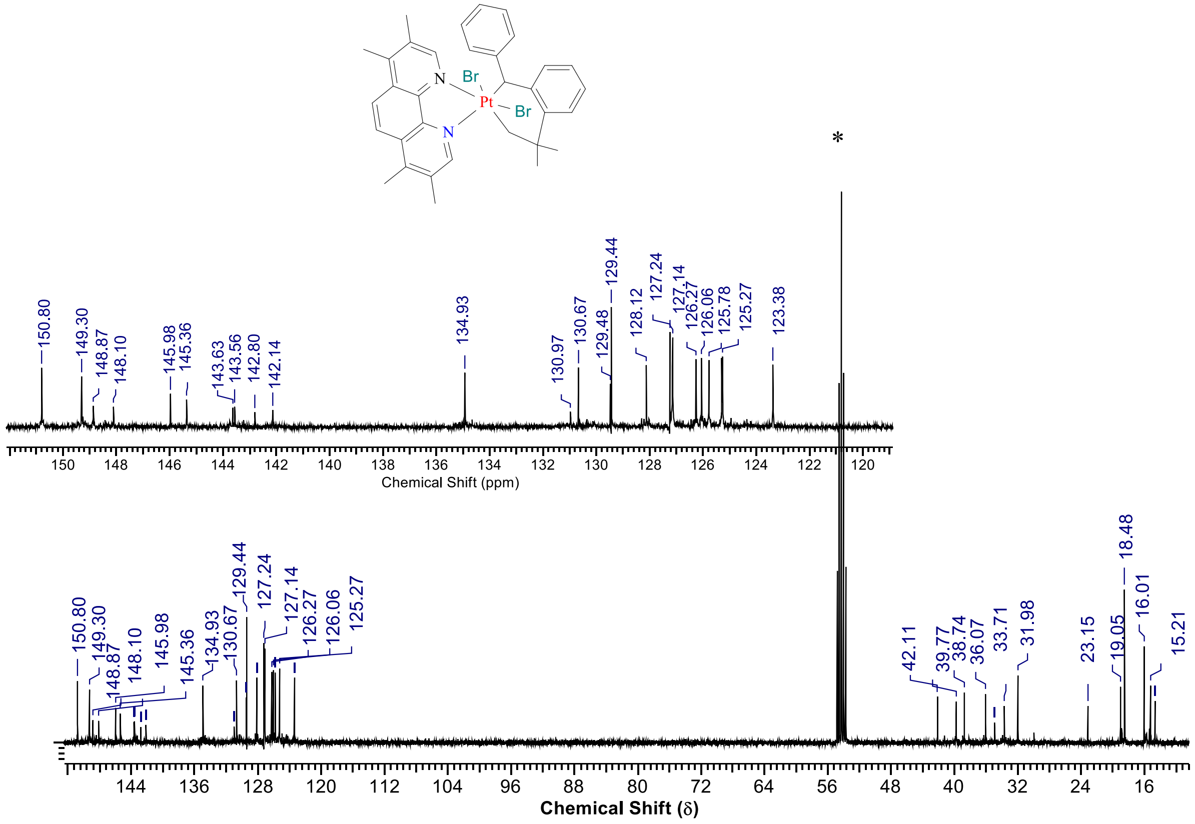

Figure S7: ^{13}C NMR spectrum of complex **4** in CD_2Cl_2.

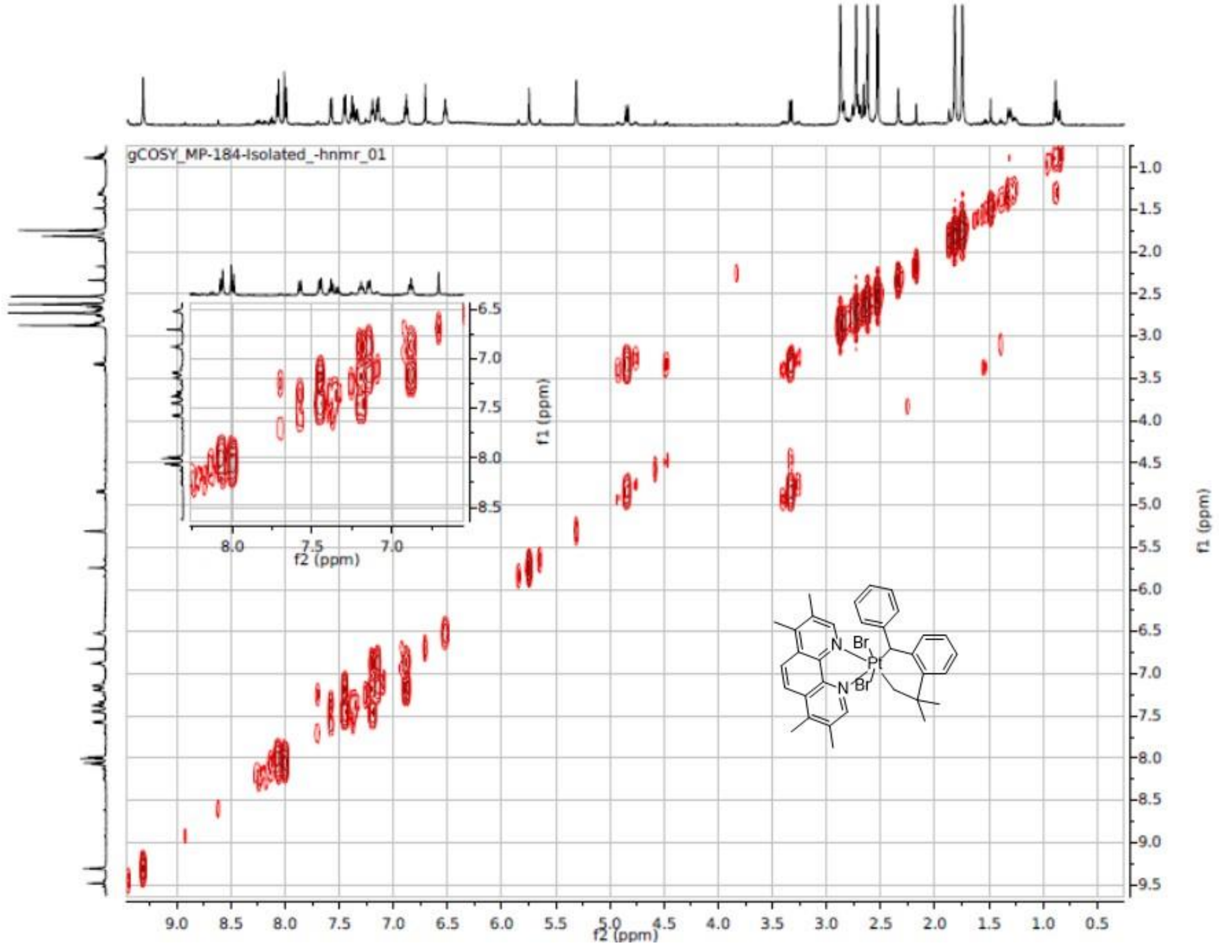

Figure S8: ^{1}H-^{1}H COSY spectrum of complex **4** in CD_2Cl_2.

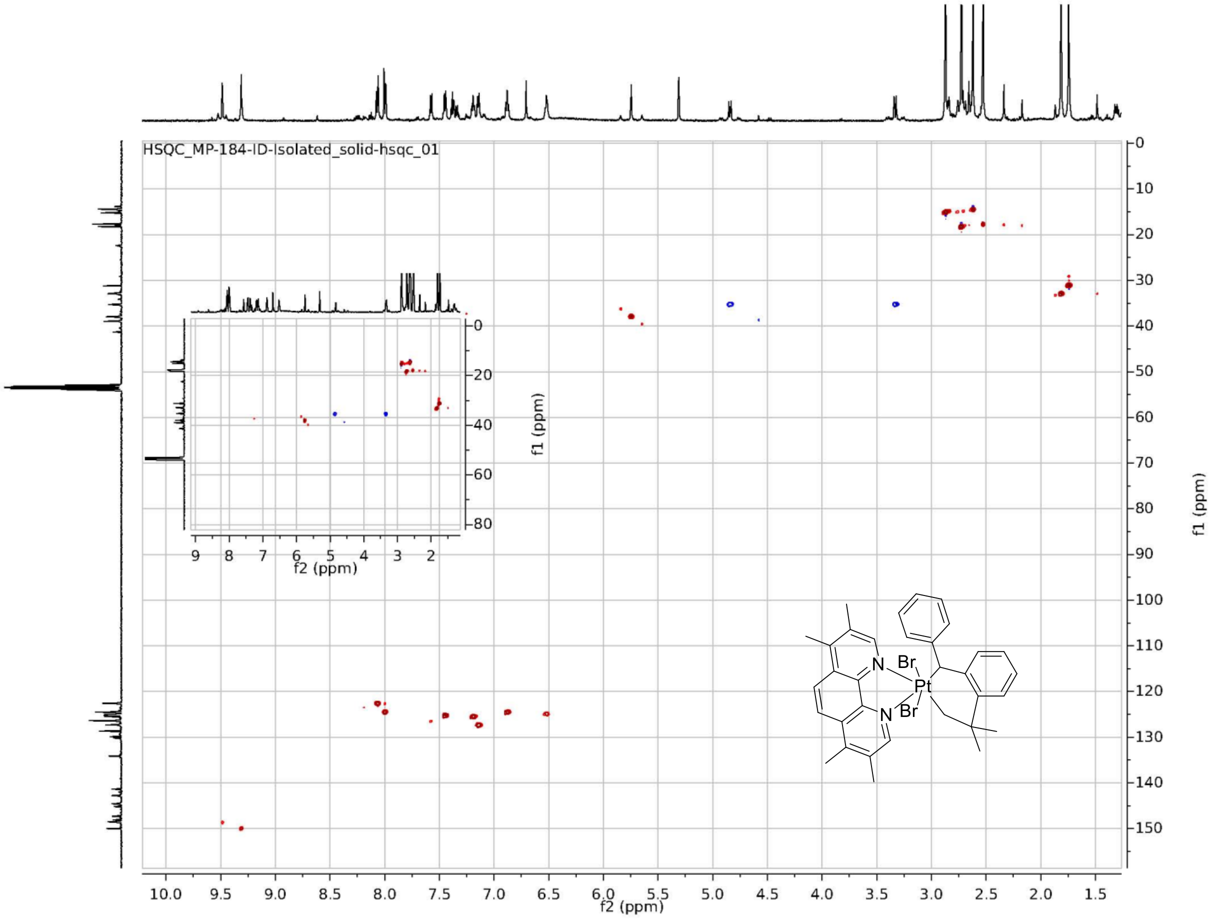

Figure S9: ^{1}H-^{13}C HSQC spectrum of complex **4** in CD_2Cl_2.

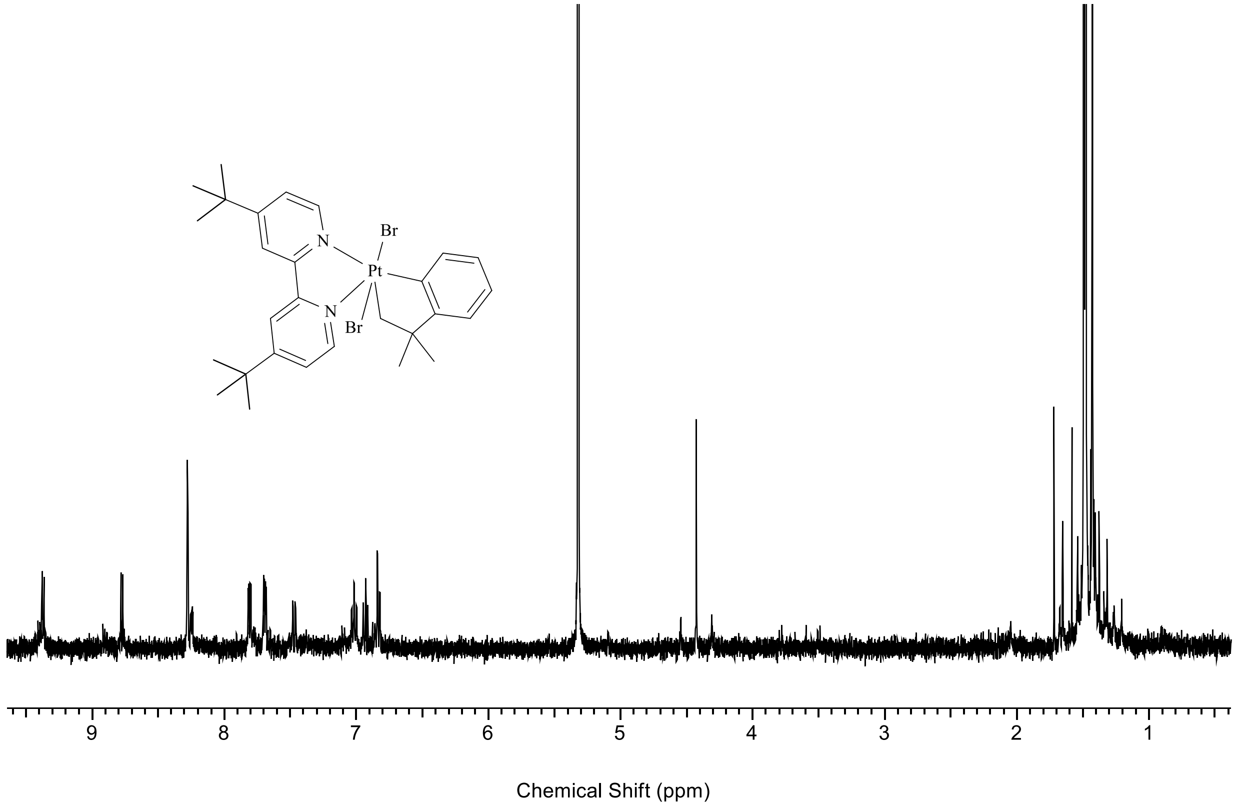

Figure S10: ^{1}HNMR spectrum of complex **7** in CD_2Cl_2.

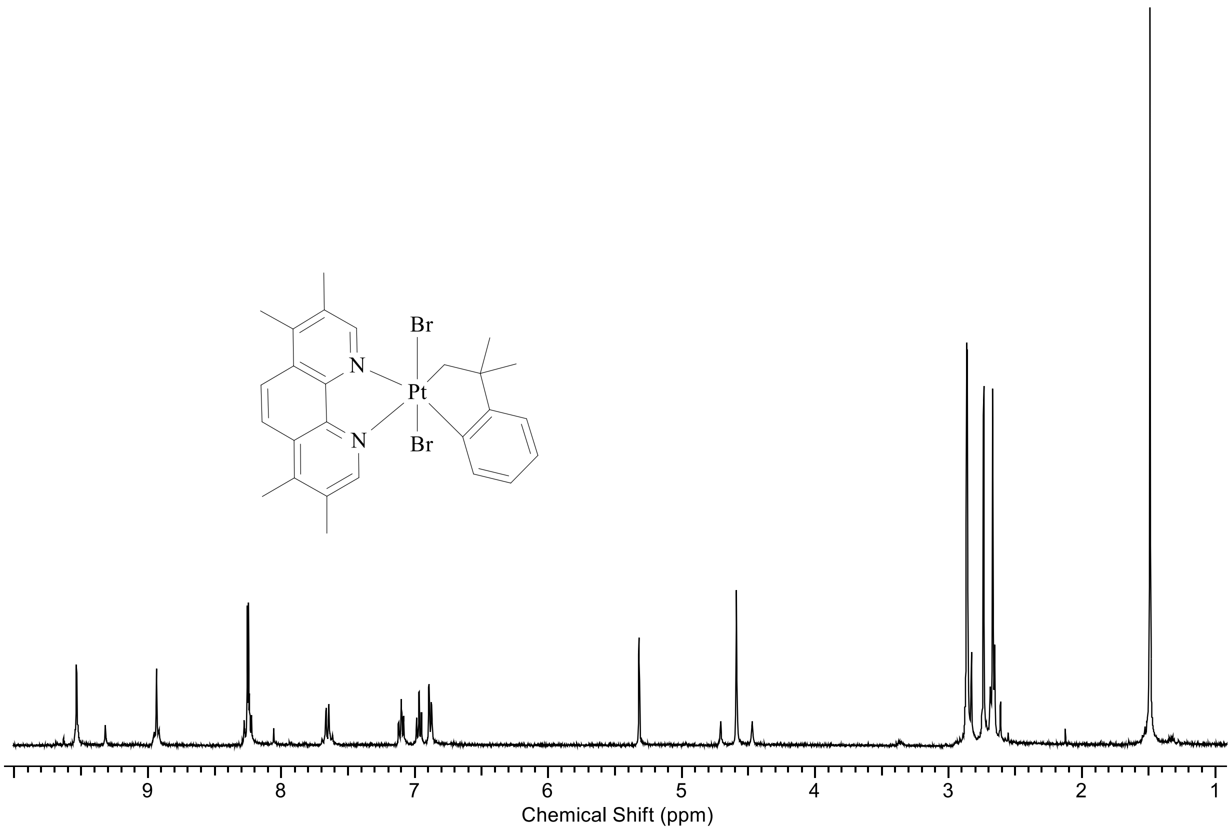

Figure S11: ^{1}HNMR spectrum of complex **8** in CD_2Cl_2.

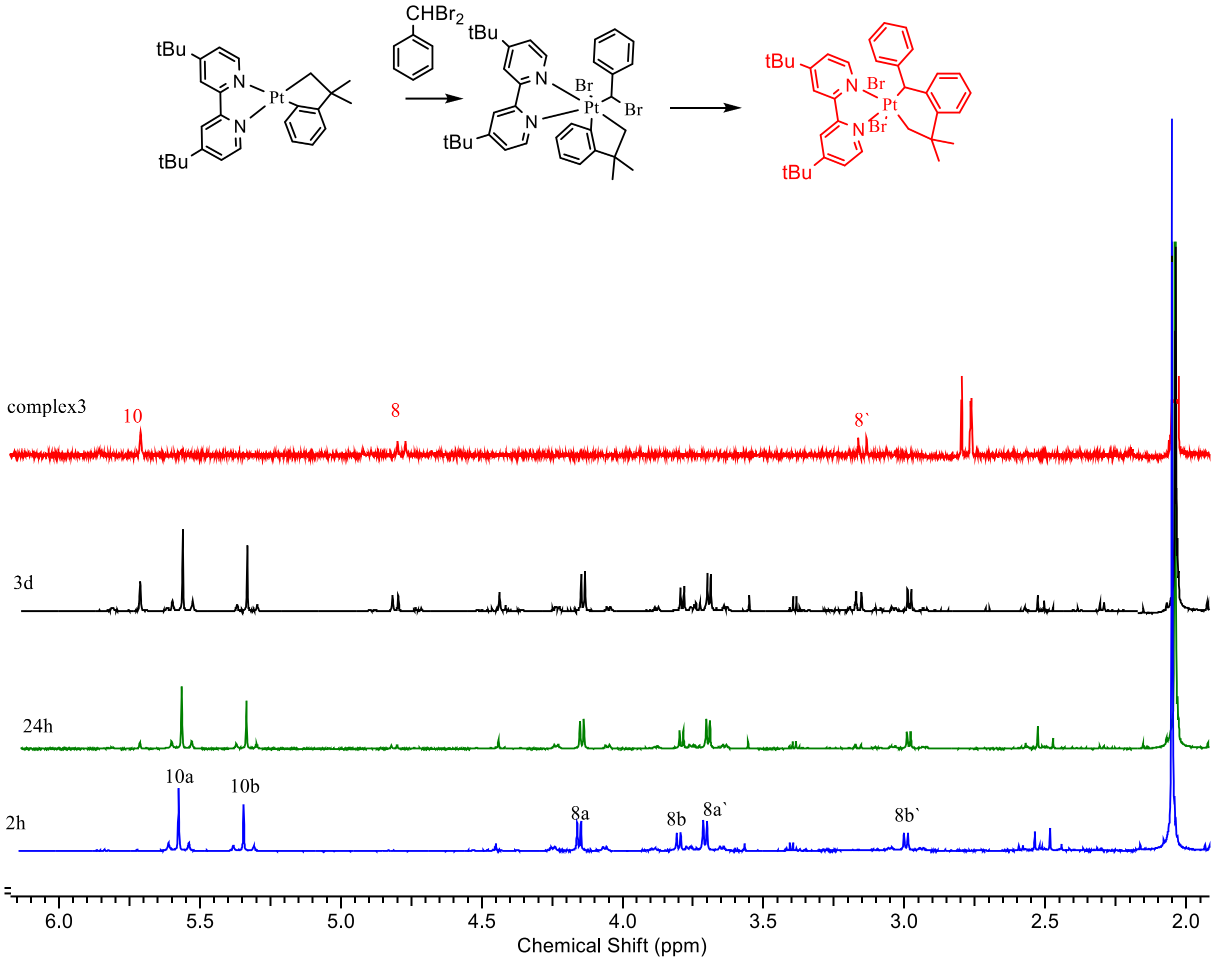

Figure S12. ^{1}HNMR spectra (Pt-CH_2 region): Monitoring of formation of complex **3** from **5**

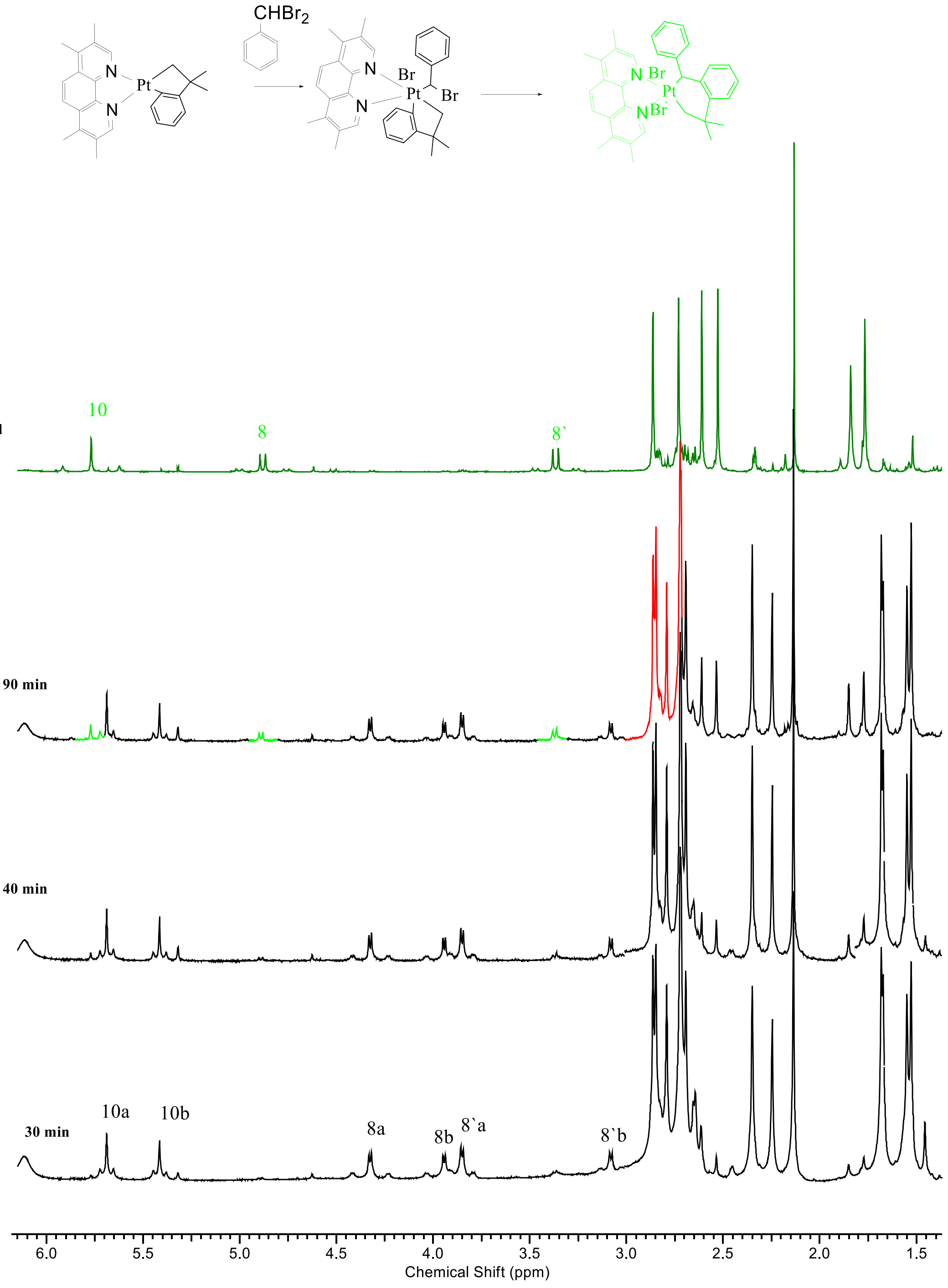

Figure S13. ^{1}H NMR spectra **(**Pt-CH_2 region): Monitoring of formation of complex **4 f**rom **6**
